# CHILDLESSNESS AND SUBJECTIVE WELL-BEING IN 88 COUNTRIES: THE ROLES OF STIGMA, DEVELOPMENT, AND REGION

**DOI:** 10.1093/geroni/igae098.1768

**Published:** 2024-12-31

**Authors:** Bussarawan Teerawichitchainan, Christine Mair, Haruka Sugimori

**Affiliations:** National University of Singapore, Singapore, Singapore; University of Maryland Baltimore County, Baltimore, Maryland, United States; National University of Singapore, Singapore, Singapore

## Abstract

Although childlessness is often hypothesized to be associated with poorer subjective well-being among aging populations, cross-national empirical evidence suggests substantial variation across countries. Yet, the reasons for this variation have yet to be identified. We hypothesize that childless older adults have lower subjective well-being in country contexts with higher stigma against childlessness, net of region, fertility, and economic development. We use multilevel modeling to analyze recent data (2017-2022) from the World Values Survey, United Nations, and World Bank, focusing on 63,067 adults aged 45+ in 88 low-middle, middle-high, and high-income countries to examine individual-level associations between childlessness and subjective well-being (life satisfaction and happiness) and moderation of country-level variables (stigma, economic development, and region). Overall, childless respondents report lower subjective well-being (life satisfaction and happiness), but these associations vary by country-level factors. Childless respondents in countries with high stigma report the lowest life satisfaction, net of fertility rate, economic development, and other factors. Happiness increases with economic development for both childless and non-childless individuals, and does not appear to be associated with stigma against childlessness. Regional differences are also evident. We discuss these findings in light of heterogeneity in the experiences of childless aging populations cross-nationally. Additionally, we highlight the potential contextualizing role of country norms and stigma when considering childlessness and well-being, particularly in family-centered or low-middle income countries.

